# Performance of ChatGPT on the Chinese Postgraduate Examination for Clinical Medicine: Survey Study

**DOI:** 10.2196/48514

**Published:** 2024-02-09

**Authors:** Peng Yu, Changchang Fang, Xiaolin Liu, Wanying Fu, Jitao Ling, Zhiwei Yan, Yuan Jiang, Zhengyu Cao, Maoxiong Wu, Zhiteng Chen, Wengen Zhu, Yuling Zhang, Ayiguli Abudukeremu, Yue Wang, Xiao Liu, Jingfeng Wang

**Affiliations:** 1 Department of Endocrine The Second Affiliated Hospital of Nanchang University Jiangxi China; 2 Department of Cardiology The Eighth Affiliated Hospital of Sun Yat-sen University Shenzhen China; 3 College of Kinesiology Shenyang Sport University Shenyang China; 4 Department of Cardiology Sun Yat-sen Memorial Hospital of Sun Yat-sen University Guangzhou China; 5 Department of Cardiology The First Affiliated Hospital of Sun Yat-sen University Guangzhou China

**Keywords:** ChatGPT, Chinese Postgraduate Examination for Clinical Medicine, medical student, performance, artificial intelligence, medical care, qualitative feedback, medical education, clinical decision-making

## Abstract

**Background:**

ChatGPT, an artificial intelligence (AI) based on large-scale language models, has sparked interest in the field of health care. Nonetheless, the capabilities of AI in text comprehension and generation are constrained by the quality and volume of available training data for a specific language, and the performance of AI across different languages requires further investigation. While AI harbors substantial potential in medicine, it is imperative to tackle challenges such as the formulation of clinical care standards; facilitating cultural transitions in medical education and practice; and managing ethical issues including data privacy, consent, and bias.

**Objective:**

The study aimed to evaluate ChatGPT’s performance in processing Chinese Postgraduate Examination for Clinical Medicine questions, assess its clinical reasoning ability, investigate potential limitations with the Chinese language, and explore its potential as a valuable tool for medical professionals in the Chinese context.

**Methods:**

A data set of Chinese Postgraduate Examination for Clinical Medicine questions was used to assess the effectiveness of ChatGPT’s (version 3.5) medical knowledge in the Chinese language, which has a data set of 165 medical questions that were divided into three categories: (1) common questions (n=90) assessing basic medical knowledge, (2) case analysis questions (n=45) focusing on clinical decision-making through patient case evaluations, and (3) multichoice questions (n=30) requiring the selection of multiple correct answers. First of all, we assessed whether ChatGPT could meet the stringent cutoff score defined by the government agency, which requires a performance within the top 20% of candidates. Additionally, in our evaluation of ChatGPT’s performance on both original and encoded medical questions, 3 primary indicators were used: accuracy, concordance (which validates the answer), and the frequency of insights.

**Results:**

Our evaluation revealed that ChatGPT scored 153.5 out of 300 for original questions in Chinese, which signifies the minimum score set to ensure that at least 20% more candidates pass than the enrollment quota. However, ChatGPT had low accuracy in answering open-ended medical questions, with only 31.5% total accuracy. The accuracy for common questions, multichoice questions, and case analysis questions was 42%, 37%, and 17%, respectively. ChatGPT achieved a 90% concordance across all questions. Among correct responses, the concordance was 100%, significantly exceeding that of incorrect responses (n=57, 50%; *P*<.001). ChatGPT provided innovative insights for 80% (n=132) of all questions, with an average of 2.95 insights per accurate response.

**Conclusions:**

Although ChatGPT surpassed the passing threshold for the Chinese Postgraduate Examination for Clinical Medicine, its performance in answering open-ended medical questions was suboptimal. Nonetheless, ChatGPT exhibited high internal concordance and the ability to generate multiple insights in the Chinese language. Future research should investigate the language-based discrepancies in ChatGPT’s performance within the health care context.

## Introduction

Artificial intelligence (AI) was initially conceptualized in 1956 [1], but it has only gained significant momentum in recent years. AI aims to replicate human intelligence and thinking processes through the use of brain-like computer systems to solve complex problems. What is most inspiring is that AI systems can be trained on specific data sets to improve prediction accuracy and tackle intricate problems [2-4], which means that one of the possible applications of AI is the ability to help doctors to rapidly search through vast amounts of medical data, enhancing their creativity and enabling them to make error-free decisions [5,6].

ChatGPT (OpenAI) is an AI model that has spurred great attention due to the revolutionary innovations in its ability to perform a diverse array of natural language tasks. By using a class of large-scale language models, ChatGPT (version 3.5) can predict the likelihood of a sequence of words based on the context of the preceding words. With sufficient training on vast amounts of text data, ChatGPT can generate novel word sequences that closely resemble natural human language and have never been observed before by other AI [7].

A study was conducted on the effectiveness of the version of generative pretrained transformer’s large-scale language model (ChatGPT, version 3.5) in passing the United States Medical Licensing Examination (USMLE). The results showed that the AI model achieved an accuracy rate of over 50% in all the tests, and in some analyses, it even surpassed 60% accuracy. It is imperative to highlight and emphasize that the study was conducted mostly using English input, and the AI model was also trained in English.

However, despite the success of AI models like ChatGPT in the English language, their performance in understanding and generating medical text in the Chinese language remains largely unexplored because ChatGPT’s ability to understand and generate text in any given language is limited by the quality and quantity of training data available in that language. Chinese is the second-most widely spoken language in the world, with more than 1.3 billion speakers globally, while the quality and quantity of Chinese language data may not be compared with English due to some reasons, such as complexity of the written characters. Thus, the performance of ChatGPT in Chinese medical information warrants further investigation.

In this study, ChatGPT’s clinical reasoning ability was evaluated by administering questions from the Chinese Postgraduate Examination for Clinical Medicine. This standardized and regulated test assesses candidates’ comprehensive abilities. The questions are textually and conceptually dense, and the difficulty and complexity of the questions are highly standardized and regulated. Additionally, this examination has demonstrated remarkable stability in raw scores and psychometric properties over the past years. Moreover, the examination comprises 43% (n=71) basic science and medical humanities, with 14% (n=23) physiology, 10% (n=17) biochemistry, 13% (n=28) pathology, and 6% (n=10) medical humanities. Clinical medicine makes up the remaining 57% (n=94), with internal medicine and surgery accounting for 37% (n=61) and 20% (n=33), respectively. Due to the examination’s linguistic and conceptual complexity, we hypothesize that it will serve as an excellent challenge for ChatGPT. By evaluating ChatGPT’s performance on this examination, we aimed to gain insights into the AI model’s potential for understanding and generating medical text in Chinese and assess its applicability in Chinese medical education and clinical practice.

## Methods

### Ethical Considerations

This study does not involve direct interaction with human participants or the collection of personal identifiable information. As a result, it falls under the category of nonhuman subject research. Therefore, no human subject ethics review approvals were required for this study. Since this study does not involve human participants or the collection of personal identifiable information, obtaining informed consent from individuals is not applicable. As this study does not involve the collection or use of personal identifiable information, privacy and confidentiality concerns are not applicable. Since this study does not involve human participants, there is no compensation provided to individuals.

### Artificial Intelligence

ChatGPT uses self-attention mechanisms and extensive training data to generate contextually relevant responses in a conversational setting. It excels in managing long-range dependencies and creating coherent replies. However, it is important to clarify that ChatGPT (version 3.5), a server-based language model, does not possess internet browsing or search functionalities. Consequently, its responses are constructed solely on abstract relationships between words or “tokens” within its neural network [7]. Furthermore, it should be noted that OpenAI released the latest version, ChatGPT (version 4), in March 2023, but the data in this study were from February 2023, when ChatGPT (version 3.5) was the most recent version.

### Input Source

The Chinese Postgraduate Examination for Clinical Medicine questions from 2022 were not officially released. However, a comprehensive set of 165 questions totaling 500 points was found on the web (Table S1 in Multimedia Appendix 1) and treated as original questions. Point values differed among question types: each case analysis question (CAQ) and multichoice question (MCQ) was worth 2 points, while common questions (CQs) were either worth 1.5 or 2 points each. All inputs fed into the ChatGPT (version 3.5) model were valid samples, not part of the training data set. This was due to the database not being updated since September 2021, predating the release of these questions. For future research convenience, the 165 questions were categorized into three types:

1. CQs (n=90): These questions are to evaluate the knowledge of basic science in physiology, biochemistry, pathology, and medical humanities. Each question has 4 choices, and the respondent should select only the correct answers. For example: “The closing time of the aortic valve during the cardiac cycle is? (A) Atrial systolic end card, (B) Rapid ejection beginning, (C) Slow ejection beginning, (D) Isovolumic diastole beginning.”
2. CAQs (n=45). It is a method used in clinical medicine to examine and evaluate patient cases. It involves an in-depth review of a patient’s medical history, presenting symptoms, laboratory and imaging results, and diagnostic findings to arrive at a diagnosis and treatment plan. There are 4 choices, and the respondent should select only the correct answers. The difference between CAQs and CQs is that CQs focus on clinical decision-making. For example: “A 38-year-old male, suffering chest pain and fever for 3 days, having a 5 years of diabetes history. Physical examination: *t*=37.6℃, right lower lung turbid knock, breathing sound is reduced. A chest X radiograph suggests a right pleural effusion. Pleural aspiration liquefaction test showed WBC 650×106/L with fine lymph Cell 90% in pleural fluid, with glucose of 3.2 mmol/L, the diagnosis for this patient is? (A) Tuberculous pleurisy, (B) Malignant pleural effusion, (C) Empyema, (D) Pneumonia-like pleural effusion.”
3. MCQs (n=30): There are 4 choices, and the respondent should select at least 2 correct answers. There is no point for choosing more or less. For example: “The structures of auditory bone conduction include? (A) Skull, (B) Round window film, (C) Ossicular chain, (D) Cochlear bone wall.”

### Scoring

Initially, the question format had to be adjusted to properly evaluate the performance of ChatGPT in the Chinese Postgraduate Examination for Clinical Medicine questions. Specifically, we included a “multichoice” or “single-choice” notation, as we found ChatGPT’s responses varied without these cues. MCQs were adjusted to state “Please choose one or more correct options,” while CQs and CAQs were altered to indicate “There is only one correct answer.” This adjustment was necessary for evaluating ChatGPT’s performance in the Chinese language.

We then compiled a data set of these examination questions along with their correct answers. To ensure validity, the answers were cross-verified with web-based resources and consultations with senior doctors. ChatGPT’s performance was then evaluated by comparing its responses to the standard answers in the data set. A high examination score would suggest that ChatGPT handled this task effectively.

In our comprehensive analysis, we also delved into examining the correlation between different question types and accuracy using the Pearson correlation coefficient as a statistical measure to investigate this relationship.

### Encoding

The structured examination questions were transformed into open-ended inquiries for better simulation of real-world clinical scenarios. Multiple-choice questions for the CAQ were removed, and ChatGPT was required to diagnose the patient’s disease and prove its reason.

Regarding the MCQs, we eliminated all the choices and did not prompt ChatGPT about the existence of multiple correct answers. The CQs were treated similarly to the MCQs. However, we encountered a distinct subset within these 3 categories that could not be processed like the other questions. This subset comprised questions that required 1 answer choice to be selected from the provided options. Therefore, these questions were converted into a special format (n=26).

For instance, an original question like, “Which can inhibit insulin secretion? (A) Increased free fatty acids in blood, (B) Increased gastric inhibitory peptide secretion, (C) Sympathetic nerve excitation, (D) Growth hormone secretion increases” was reformatted as “Can an increase in free fatty acids in the blood, an increase in gastric inhibitory peptide secretion, an increase in sympathetic nerve excitation, or an increase in growth hormone secretion inhibit insulin secretion?” This encoding strategy was applied across all 3 subgroups.

Additionally, to mitigate potential memory retention bias, we commenced a new chat session for each query. This process of reformatting questions, presenting them to ChatGPT, and initiating new sessions for each question constituted our methodology for evaluating ChatGPT’s performance using the data set. The clarity of this process should address the concerns raised in the comment about the lack of understanding of the way we used the data set for evaluation.

### Adjudication

To assess ChatGPT’s performance thoroughly, 2 physicians independently scored AI outputs for accuracy, concordance, and insight using predefined criteria (Table S2 in Multimedia Appendix 1). These physicians were not aware of each other’s evaluations. To familiarize the physicians with the scoring system, a subset of 20 questions was used for training, during which the physicians were unblinded to each other’s assessments.

ChatGPT’s responses were classified into 3 categories under the accuracy parameter: accurate, inaccurate, and indeterminate. “Accurate” responses were those where ChatGPT provided the right answer, while “inaccurate” encompassed instances of no answer, an incorrect response, or multiple answers containing incorrect options. “Indeterminate” responses were those where the AI output did not present a definitive answer, suggesting insufficient information to make a selection.

Concordance was determined by whether ChatGPT’s explanation affirmed its provided answer, with discordance occurring if the explanation contradicted the answer. We defined valuable insights as unique text segments within the AI’s explanations meeting specific criteria: they were nondefinitional, nonobvious, valid, and unique. These insights required additional knowledge or deductions beyond the input question, provided accurate clinical or numerical information, and potentially eliminated multiple answer choices with a single insight.

**Figure 1:**
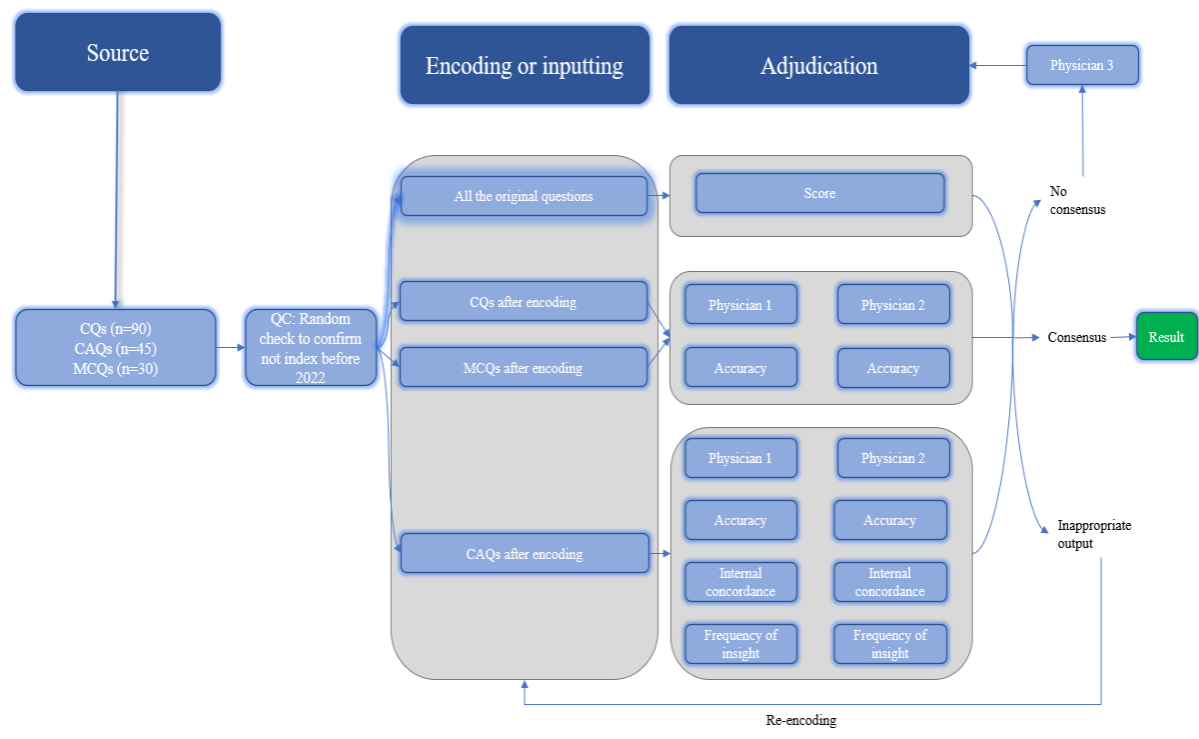
Schematic of workflow for sourcing, encoding, and adjudicating results. The 165 questions were categorized into 3 types: CQ, CAQ, and MCQ, and each question was assessed for its score. The accuracy of the CQ and MCQ questions was evaluated, while the MCQ questions were also assessed for the accuracy, concordance, and frequency of insights. The adjudication process was carried out by 2 physicians, and in case of any discrepancies in the domains, a third physician was consulted for adjudication. Additionally, any inappropriate output was identified and required re-encoding. CAQ: case analysis question; CQ: common question; MCQ: multichoice question.

To mitigate potential within-item anchoring bias, the adjudicators first evaluated the accuracy for all items, followed by concordance. In case of discrepancies in domain assessments, a third physician adjudicator was consulted. This third-party intervention was required for 11 items (n=11, 7% of the data set). We used the Cohen κ statistic to evaluate the interrater agreement between the physicians for the questions (Table S3 in Multimedia Appendix 1). A schematic overview of the study protocol is presented in Figure 1 to provide a clearer understanding of our methodology.

## Results

### ChatGPT Performs Poor Toward the Original Questions

After inputting the original questions into ChatGPT and collecting their answers, ChatGPT received a score of 153.5 out of 300, which means that it only obtained 51.2% of the total points on the test. This score is much lower than expected but slightly higher than the passing threshold (129/300) defined by official agencies.

**Figure 2:**
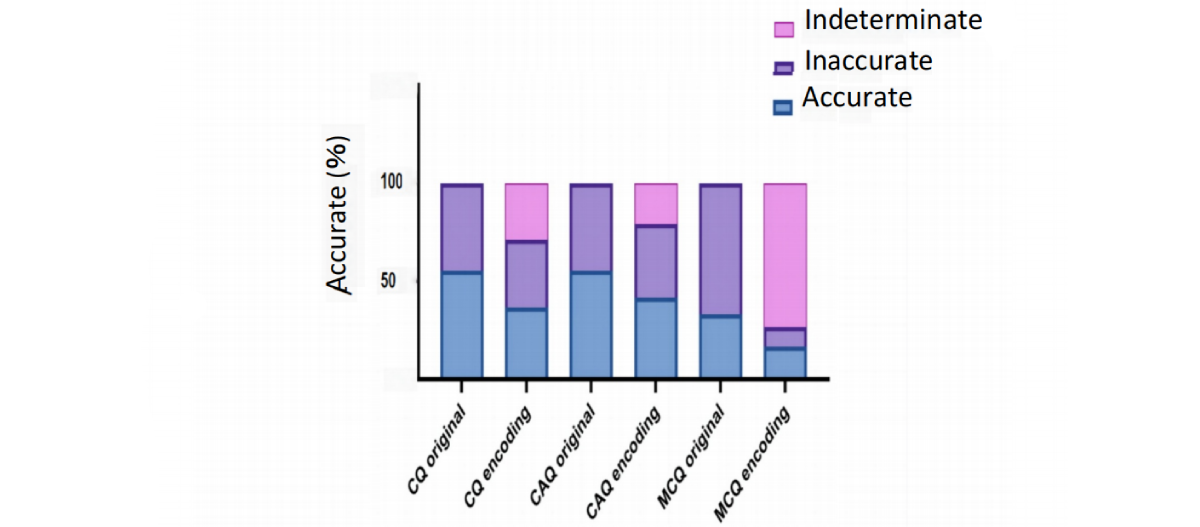
Accuracy of ChatGPT on Chinese Postgraduate Examination for Clinical Medicine before and after encoding. For the subgroups CQ, CAQ, and MCQ before encoding, AI output was compared with the standard answer key. For the subgroups CQ, CAQ, and MCQ after encoding, AI outputs were adjudicated to be accurate, inaccurate, or indeterminate based on the scoring system provided in Table S2 in Multimedia Appendix 1 data. It demonstrates the different accuracy distribution for inputs between the before and after encoding. AI: artificial intelligence; CAQ: case analysis question; CQ: common question; MCQ: multichoice question.

Among 3 subgroups of questions, the evaluation revealed that of a total of 90 CQs, ChatGPT only provided 50 (56%, 95% CI 45%-66%) correct answers. Similarly, of 45 CAQs, ChatGPT provided 25 (56%, 95% CI 41%-70%) correct answers. Furthermore, of 30 MCQs, ChatGPT provided 10 (33%, 95% CI 16%-50%) completely accurate answers (Figure 2). These results suggest that ChatGPT’s ability to resolve medical problems in Chinese needs to be improved.

Additionally, we have noticed a Pearson correlation coefficient value of approximately 0.228. This finding suggests a relatively weak correlation between the different question types and the accuracy of the responses.

### ChatGPT Performs Worse on Encoded Questions Compared to the Original Questions

We encoded questions from the Chinese Postgraduate Examination for Clinical Medicine and inputted them into ChatGPT, which simulates scenarios where a student answers a common medical question without any choices or a doctor tries to diagnose a patient based on multimodal clinical data (ie, symptoms, history, physical examination, and laboratory values). ChatGPT’s accuracy for all questions was 31.5%. Among the 3 subgroups, namely, CQs, MCQs, and CAQs, the accuracy was 42%, 37%, and 17%, respectively (Figure 2). Compared to the original questions, the accuracy of the encoding questions decreased by 19%, 17%, and 14% for CQs, MCQs, and CAQs, respectively, which demonstrates that the ability of ChatGPT to answer the open-ended questions in Chinese is a shortcoming. During the adjudication stage, there was substantial agreement among physicians on prompts in all 3 subgroups (κ ranged from 0.80 to 1.00).

### ChatGPT Demonstrates High Internal Concordance

**Figure 3:**
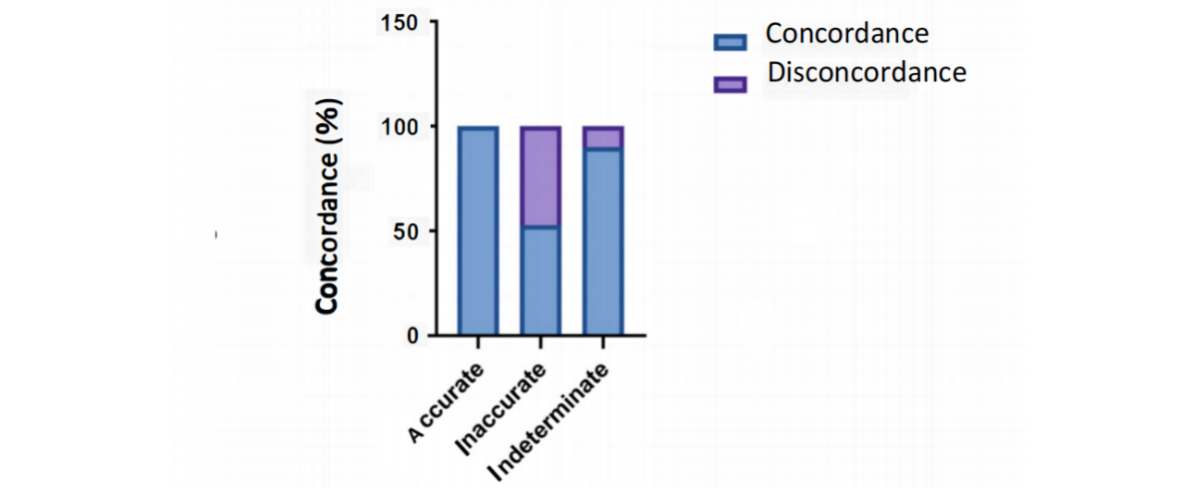
Concordance of ChatGPT on Chinese Postgraduate Examination for Clinical Medicine after encoding. For the subgroup “case analysis question,” artificial intelligence outputs were adjudicated to be concordant and discordant based on the scoring system provided in Table S2 in Multimedia Appendix 1 data. It demonstrates concordance rates stratified between accurate, inaccurate, and indeterminate outputs across all the case analysis questions.

Concordance, which is a measure of the level of agreement or similarity between the option selected by AI and its subsequent explanation, was also taken into consideration. The results showed that ChatGPT achieved 90% concordance across all questions, and this high concordance was maintained across all 3 subgroups (Figure 3). Additionally, we analyzed the concordance difference between correct and incorrect answers and found that concordance among accurate responses was perfect and significantly greater than among inaccurate responses (n=52, 100% vs n=113, 50%; *P*<.001; Figure 3). These findings suggest that ChatGPT has a high level of answer-explanation concordance in Chinese, likely due to its strong internal consistency in its probabilistic language model.

### ChatGPT Shows Multiple Insights Toward the Same Questions

**Figure 4:**
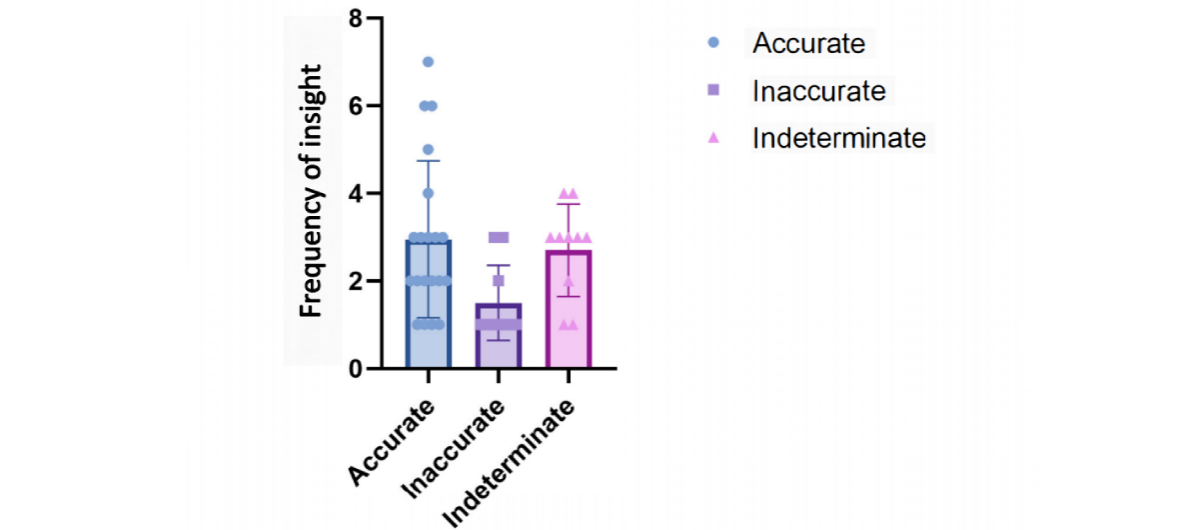
The frequency of insights of ChatGPT on Chinese Postgraduate Examination for Clinical Medicine after encoding. For the subgroup “case analysis question,” artificial intelligence outputs were adjudicated to count the frequency of insights it offered. It demonstrates the frequency of insights stratified between accurate, inaccurate, and indeterminate outputs, across all the case analysis questions.

Another evaluation index considered was the frequency of insights generated by the AI model that quantifies the quantity of insights produced. After evaluating the score, accuracy, and concordance of ChatGPT, its potential was investigated to enhance medical education by augmenting human learning. We analyzed the frequency of insights provided by ChatGPT. Remarkably, ChatGPT generated at least 1 significant insight in 80% (n=132) of all questions (Figure 4). Moreover, the analysis revealed that the accuracy response had the highest frequency of insights with an average of 2.95. The indeterminate response followed closely behind with an average of 2.7, while the inaccurate response had a lower frequency of insights with an average of 1.39 (Figure 4). The high frequency of insights in the accurate group suggests that it may be feasible for a target learner to acquire new or remedial knowledge from the ChatGPT AI output.

## Discussion

### Major Findings

To evaluate ChatGPT’s problem-solving capabilities and assess its potential for Chinese medical education integration, its performance on the Chinese Postgraduate Examination for Clinical Medicine was tested. We had two major findings: (1) the score of ChatGPT needs to be improved when facing questions asked in the Chinese language and (2) there is still potential for this AI to generate novel performance that can assist humans due to the high concordance and the frequency of insights. This is the first study to assess the performance of ChatGPT in medical care and clinical decisions in Chinese.

### ChatGPT’s Performance Needs Improvement for Medical Questions in Chinese

A recent study showed that ChatGPT (version 3.5) performed with an accuracy rate of over 50% across all examinations and even exceeded 60% accuracy in some analyses when facing the USMLE [7]. Our results indicate that ChatGPT exhibited moderate accuracy in answering open-ended medical questions in Chinese, with an accuracy of 31.5%. Given the differences between English and Chinese inputs, it suggests that ChatGPT requires further improvement in answering medical questions in the Chinese language.

We sought to understand why there is a significant discrepancy between the performance of ChatGPT on Chinese and English language examinations. To investigate this, we asked the ChatGPT for the reasons, it explains that the training data used to train AI in different languages may be different, and the algorithms used to process and analyze text may vary from language to language (data not shown). Therefore, even for the same question, the output generated may vary slightly based on the language and the available language-based data.

Upon analyzing the results of this research, we found that the accuracy of ChatGPT was lowest for MCQs, followed by CQs and CAQs. The lower accuracy on MCQs may be due to the model being undertrained on the input as well as the MCQ samples being significantly less than those of single-choice questions. On the other hand, the CAQs may have extensive training compared to MCQs and are similar in type to the USMLE question.

Furthermore, we noticed that high accuracy outputs were associated with high concordance and a high frequency of insight, whereas poorer accuracy was linked to lower concordance and a lack of insight. Thus, it was hypothesized that inaccurate responses were primarily driven by missing information, which could result in reduced insight and indecision in the AI, rather than an overcommitment to an incorrect answer [7]. The results indicate that enhancing the database and providing additional training with Chinese questions could substantially improve the performance of the model.

### Challenges of AI in Future Applications

Despite the promising potential of AI in medicine, it also poses some challenges. Standards for using AI in health care still need to be developed [8,9], including clinical care, quality, safety, malpractice, and communication guidelines. Furthermore, the implementation of AI in health care requires a shift in medical culture, which poses a challenge for both medical education and practice. Additionally, ethical considerations must be taken into account, such as data privacy, informed consent, and bias prevention, to ensure that AI is used ethically and for the benefit of patients. Surprisingly, a recently launched AI system for autonomous detection of diabetic retinopathy carries medical malpractice and liability insurance [10].

### Prospective of AI

AI is a rapidly growing technology. At the time of writing, ChatGPT (version 4) has been released with significant improvements. Numerous practical and observational studies have demonstrated the versatile role of AI in almost all medical disciplines and specialties, particularly in improving risk assessment [11,12], data reduction, clinical decision support [13,14], operational efficiency, and patient communication [15,16]. We anticipate that advanced language models such as ChatGPT are reaching a level of maturity that will soon have a significant impact on clinical medicine, enhancing the delivery of personalized, compassionate, and scalable health care.

A comparison of ChatGPT’s performance with other AI models, particularly in the context of Chinese language performance, could yield more comprehensive insights and underscore the unique challenges of using AI in diverse linguistic landscapes.

However, this was primarily due to the fact that AI models that focus on other aspects, while enhancing medical education and achieving promising results in medical question answering, are mostly developed and evaluated using English language data sets. This limitation restricts their applicability for performance comparisons in the context of the Chinese language.

### Limitations

One limitation of this research is the small sample size. We only accessed 165 samples to qualify its accuracy and 30 CAQs to qualify its concordance and frequency of insight due to the limitations of the data, which focused solely on the diagnosis of the patient. Furthermore, the clinical situation is more complicated than the test, and larger and deeper analyses were needed. Finally, bias and error were inevitable in human adjudication, although there was a good interrater agreement between the physicians for the adjudication.

Moreover, comparing ChatGPT’s performance with other AI models, especially in the context of Chinese language, can provide valuable insights and highlight the distinctive challenges associated with leveraging AI in diverse linguistic environments.

One notable factor contributing to this need for comparison is the prevalence of AI models such as Bidirectional Encoder Representations from Transformers, CLUE-Med, and MedQA that have made significant contributions to medical education and demonstrated promising outcomes in medical question answering. However, these models have predominantly been developed and assessed using English language data sets. This particular limitation hampers their suitability for conducting performance assessments within the Chinese language domain.

### Conclusions

In conclusion, although the ChatGPTs got a score over the passing score in the Chinese Postgraduate Examination for Clinical Medicine, the performance was limited when presented with open-ended questions. On the other hand, ChatGPT demonstrated a high level of internal concordance, which suggests that the explanations provided by ChatGPT support and affirm the given answers. Moreover, ChatGPT generated multiple insights toward the same questions, demonstrating its potential for generating a variety of useful information. Further prospective studies are needed to explore whether there is a language-based difference in the performance of medical education settings and clinical decision-making, such as Chinese and minority languages.

## Appendix

Multimedia Appendix 1 Original questions, adjudication criteria for accuracy and concordance, and κ statistic for interrater agreement between adjudicating physicians.

## Abbreviations

AI: artificial intelligence
CAQ: case analysis question
CQ: common question
MCQ: multichoice question
USMLE: United States Medical Licensing Examination

## Data Availability

All data generated or analyzed during this study are included in this published paper (and Multimedia Appendix 1).
