# Supplementary material for: Performance of ChatGPT on the Chinese Postgraduate Examination for Clinical Medicine: Survey Study
Source: JMIR Med Educ. 2024 Feb 9;10:e48514. doi: 10.2196/48514 (PMC10891494; doi:10.2196/48514)
Supplement: Multimedia Appendix 1 [file mededu_v10i1e48514_app1.pdf]

Table S1： The original questions end at 2023

The raw data file can be accessed at the following URL:

https://drive.google.com/file/d/17_1mGKbN5MLoK6yZmSL363Q1wjIJB3wO/view?usp=share_link

Table S2. Adjudication criteria for accuracy and concordance

Accurate:1. Provide the answer accurately.<br>2.When the judge determines that there is not a unique answer, the AI outputs multiple choices, among which contains the correct answer, and the other options are also completely correct.	Concordant: Explaining affirms the answer
Inaccurate 1. No answer is provided<br>2.An incorrect answer is provided<br>3.Multiple answers are provided, among which there is an incorrect answer, even if the correct answer is included.	Discordant: Explanations are contradictory.
Indeterminate: 1.AI output is not a single answer election.<br>2.When the judge determines that there is a unique answer, AI output provides multiple choices.<br>3.AI believes that there is not enough information."	

Table S3. κ statistic for interrater agreement between adjudicating physicians.

	Accuracy		Concordance	
	Cohen κ	n	Cohen κ	n
CAQ	0.559	45	0.444	45
MCQ	0.795	30		
CQ	0.825	90		
